# The Buffalo Medical Library Association

**Published:** 1884-11

**Authors:** 


### The Buffalo Medical Library Association.

The Buffalo Library Association have issued the following circular to its members and the profession, to which we direct the attention of our readers. The value of a medical library, always open and accessible for study and reference, is acknowleged by every educated physician, and the enterprise of the directors in changing the rules so that journals can be circulated among its members deserves special encouragement. The library rooms are open daily from 2 to 9 P. M. All the principal journals of America and England, and several of the leading journals of Germany and France, are found on the library table. Journals devoted to special departments are also taken by the association. It is arranged for members who are unable to visit the library rooms to notify the librarian the special journal, with number and date, they desire, with postage enclosed, and if the journal is in, it will be sent by mail. The dues are five dollars per year.

These provisions are exceedingly liberal and will accommodate the convenience and wishes of all. They signify a very liberal and progressive policy in the present management of the association, which, we trust, will bring forth generous patronage from the profession.

At a meeting of the directors, held August 6th, a committee was appointed to consider if it was desirable to change the rules governing the library, so as to allow the journals taken to be circulated among the members. They were also empowered to act in the matter according to their judgement.

It is decided by the committee to make a fair trial of this method from October 1st to January 1st. If it meets the wishes and wants of the members it will be continued.

It is earnestly requested that none of the members will enter into any arrangement among themselves to circulate to one another any periodical, and also that no member will take advantage of any opportunity that may present to regularly get control of journals when they first arrive. Such a breach of good faith will defeat the proposed change.

M. D. Mann,

C. C. Frederick,

W. D. Granger,

*Committee.*

Library, 396 Pearl street.

August 16, 1884.

RULES GOVERNING THE CIRCULATION OF PERIODICALS.

Rule 1. A list of members who are entitled to take out periodicals will be kept posted in the library room. No one, unless his name is on the list, will be allowed this privilege.

Rule 2. Any member whose name is not on the list must apply to the librarian, who shall be the judge, and decide if it is to be added.

RULE 3. Any member who has paid the yearly dues, or signed an agreement to pay them on either April 1st, July 1st, or October 1st, shall have his name put on the list.

RULE 4. Each number will be plainly marked with the date at which it can be taken out. As a rule, a weekly will remain on the library table two weeks, and a monthly or quarterly one month.

RULE 5. A member will be entitled to but one periodical at any one time, which must be returned; and if any fines are owing, they must be paid before he shall be entitled to another publication.

RULE 6. Weekly journals may be kept three days, and a monthly or quarterly one week. They may be returned by mail, but must be placed in the mail the day before they are due, the postage fully paid, and the name of the sender placed on the wrapper.

RULE 7. A fine of ten cents a day will be due for every day a periodical is retained beyond the time allowed. A box will be provided in the library to receive the fine, with the name of the person who pays it.

RULE 8. Periodicals kept more than one week will be sent for, and the cost of sending charged to the member.

RULE 9. All fines not paid within a week will be collected by the librarian, and the cost of collecting charged.

RULE 10. All periodicals shall be taken out in person, and not by an agent. He shall enter in a book, to be kept for that purpose, the date, his name, and the number and name of the periodical; on its return, the date, and the amount of fines, if there are any.

RULE 11. If a periodical is lost, it shall be replaced by the member who drew it.

RULE 12. Great care must be used not to soil the publication, as it is kept by binding. If a number is soiled by undue carelessness, the librarian may require a new one to be furnished.

RULE 13. Each member taking a periodical is placed upon his honor to fully observe these rules in their spirit and letter. The Board reserves the right to erase the name of any person who shall willfully and grossly violate these rules.

